# Status of unwanted pregnancy and contraceptive needs among women of childbearing age in Chengdu City: a cross-sectional study

**DOI:** 10.3389/fgwh.2026.1759010

**Published:** 2026-04-07

**Authors:** Limei Wu, Jiang Li, Zhanglu Wang, Ye Liu, Xi Huang, Fan Xu, Li Li

**Affiliations:** 1 Department of Obstetrics and Gynecology, West China School of Medicine, Sichuan University, Sichuan University Affiliated Chengdu Second People's Hospital, Chengdu Second People's Hospital, Chengdu, Sichuan, China; 2 Department of Clinical Laboratory, China-Japan Friendship Hospital, Beijing, China; 3 Department of Obstetrics and Gynecology, Jianyang Maternal and Child Health Hospital, Jianyang Women and Children Hospital, Jianyang, Sichuan, China; 4 Department of Evidence Based Medicine and Social Medicine, School of Public Health; Sichuan Provincial Key Laboratory of Philosophy and Social Science For Intelligent Medical Care and Elderly Health Management; Chengdu Medical College, Chengdu, Sichuan, China; 5 Department of Gynecology, The People's Hospital of Kaizhou District, Chongqing, China

**Keywords:** contraception, contraceptive needs, pregnancy, reproductive age, unwanted pregnancy

## Abstract

The aim of this work was to assess the prevalence and associated factors of unwanted pregnancy and explore the contraceptive needs among women of childbearing age in the main districts of Chengdu. A questionnaire survey was administered in five districts of Chengdu following a stratified two-stage sampling technique. We performed descriptive, bivariate, and multivariate statistical analyses. A total of 1,289 women aged 15–44 years were randomly selected, of whom 1,230 completed the survey. Among respondents, 24.1% had experienced unwanted pregnancy. Married women had 1.665 times the odds of unwanted pregnancy compared with unmarried women, but the odds of unwanted pregnancy were 0.528 times as high for women aged 21–25 years compared with those aged ≤20 years. Women with a university or higher level of education were less likely to report unwanted pregnancy, while those with a male partner from a rural area were more likely to report unwanted pregnancy. Furthermore, women whose male partners had a junior college, university, or higher education background were less likely to report unwanted pregnancy. More than half of the respondents had never received contraceptive education. Nearly three in five reported inadequate contraceptive education, and 70.41% expressed a strong willingness to accept professional contraceptive guidance. These findings highlight that being married and having a male partner from the village are risk factors for unwanted pregnancy, while the age group 21–25 years and having a college or higher level of education are protective factors for unwanted pregnancy. Reproductive health guidance and policies are needed to reduce the rates of unwanted pregnancy in Chengdu.

## Introduction

Unwanted pregnancy refers to a pregnancy that a woman did not desire at that time or ever in the future (1). It is often the outcome of non-use, inconsistent use, or incorrect use of effective family planning methods. According to World Health Organization reports, 75 million out of 175 million registered pregnancies were unwanted worldwide in 2019 (2). In East Asia, approximately 58 unwanted pregnancies occur annually per 1,000 women, with 61% ending in an abortion (3).

Unwanted pregnancy is considered a significant public health concern. It poses a risk to both maternal and child health due to lack of family support, contributes to increased maternal mortality, is a major obstacle to the improvement of reproductive and sexual health. In recent times, a broad range of effective contraceptive methods have become available. However, misunderstandings about the availability and safety of emergency contraception lead to underuse. Therefore, despite the availability of multiple, effective contraceptive methods, the global rate of unwanted pregnancy remains high (4). Women need access to accurate information about these different methods in order to choose an option that aligns with their individual needs regarding lifestyle, family planning, risk factors, and non-contraceptive benefits (5).

Chengdu is a new first-tier city in China with a large number of young residents. There are presently more than 4.5 million women of childbearing age in the city, comprising one-fifth of the total population. To the best of our knowledge, there have been no studies investigating the prevalence and associated factors of unwanted pregnancy in this region. Therefore, the main objectives of this study were to (i) estimate the prevalence of unwanted pregnancy among women aged 15–44 years in five main districts of Chengdu; (ii) examine the associations between sociodemographic/behavioral characteristics and unwanted pregnancy; (iii) identify contraceptive guidance needs among women of childbearing age; and (iv) provide suggestions for the further development and provision of high-quality family planning services.

## Materials and methods

### Ethics statement

Ethics approval was obtained from the Chengdu Second People's Hospital Ethics Committee. The purpose and nature of the study were explained to all participants, emphasizing that participation was voluntary and they could withdraw at any time without any consequences or loss of privileges. Those who agreed to take part in the study provided written informed consent. For minors, consent was obtained from their guardians. Women who could not read were asked to provide thumbprint consent after the form was read aloud to them, emphasizing that participation was voluntary and they could withdraw at any time without any consequences. To ensure privacy and confidentiality, the self-administered questionnaire was programmed online and accessed via a QR code. Data security was ensured through restricted access permissions and confidentiality agreements.

### Study design and setting

A two-stage stratified sampling method was used to select women of childbearing age from the five main districts of Chengdu (Jinjiang, Qingyang, Chenghua, Jinniu, and Wuhou) between 12 April 2024 and 23 June 2025. With the help of community staff, respondents completed the online questionnaire.

### Study population

Based on an average contraceptive compliance rate of 58.07% (6), and considering a non-response rate of 10% and questionnaire inefficiency of 10%, the minimum required sample size was 470. The precision used in the sample size calculation was a margin of error of ±5% with a 95% confidence level. A total of 1,289 questionnaires were sent out, and 1,230 valid responses (95.4%) were included after excluding missing items, inconsistent answers, and vague answers of key variable values. Therefore, the sample size of this study met the requirements.

### Inclusion and exclusion criteria

The inclusion criteria were women of childbearing age (15–44 years), who were sexually active, willing to participate voluntarily, and residing in the five main districts of Chengdu. The exclusion criteria included women younger than 14 years or older than 44 years, those without prior sexual activity, or women who were unwilling to participate in this survey.

### Sampling method

The survey employed a stratified two-stage sampling technique in order to ensure regional representativeness. First, the survey was conducted in the five main districts of Chengdu. All women of childbearing age (15–44 years old) in these areas were selected as survey subjects. Chengdu has more than 8,700 communities, with a total population of approximately 6 million, including about 860,000 married women of childbearing age (excluding unmarried women who are sexually active). Computer-generated random numbers were used to select target communities using a cluster sampling method. Within each selected community residents' committee, simple random sampling was conducted using ID numbers to select 10 individuals as survey subjects, with an additional 3–5 alternates. When the randomly sampled population did not meet the inclusion criteria, alternates were used. With the help of the community residents' committee, the selected individuals were contacted by phone.

### Questionnaire

As none of the previously existing questionnaires addressed all of the items needed for our study, we developed a study-specific questionnaire divided into two parts: unwanted pregnancy and contraceptive demand. The self-administered questionnaire was programmed online (https://www.wjx.cn/) by scanning a predetermined QR code. We used a secure questionnaire survey platform and limited the scope of data access through access permissions to reduce the risk of information leakage. Moreover, all participants signed confidentiality agreements.

### Statistical analysis

Simple descriptive, bivariate, and multivariate statistical analyses were performed in this study. Descriptive analysis was used to describe the frequency and percentage distribution of variables. Logistic regression was employed because the dependent variable (unwanted pregnancy) was measured as a binary factor. The results of the regression analysis are presented as odds ratios (ORs), along with their corresponding 95% confidence interval (CI), indicating the precision and significance of the reported ORs. An OR of less than 1 denotes lower odds of unwanted pregnancy, while an OR greater than 1 indicates higher odds. Inherent sample weight was applied, and all analyses were conducted using STATA version 13.0 software.

## Results

### Basic subject information

**Figure 1:**
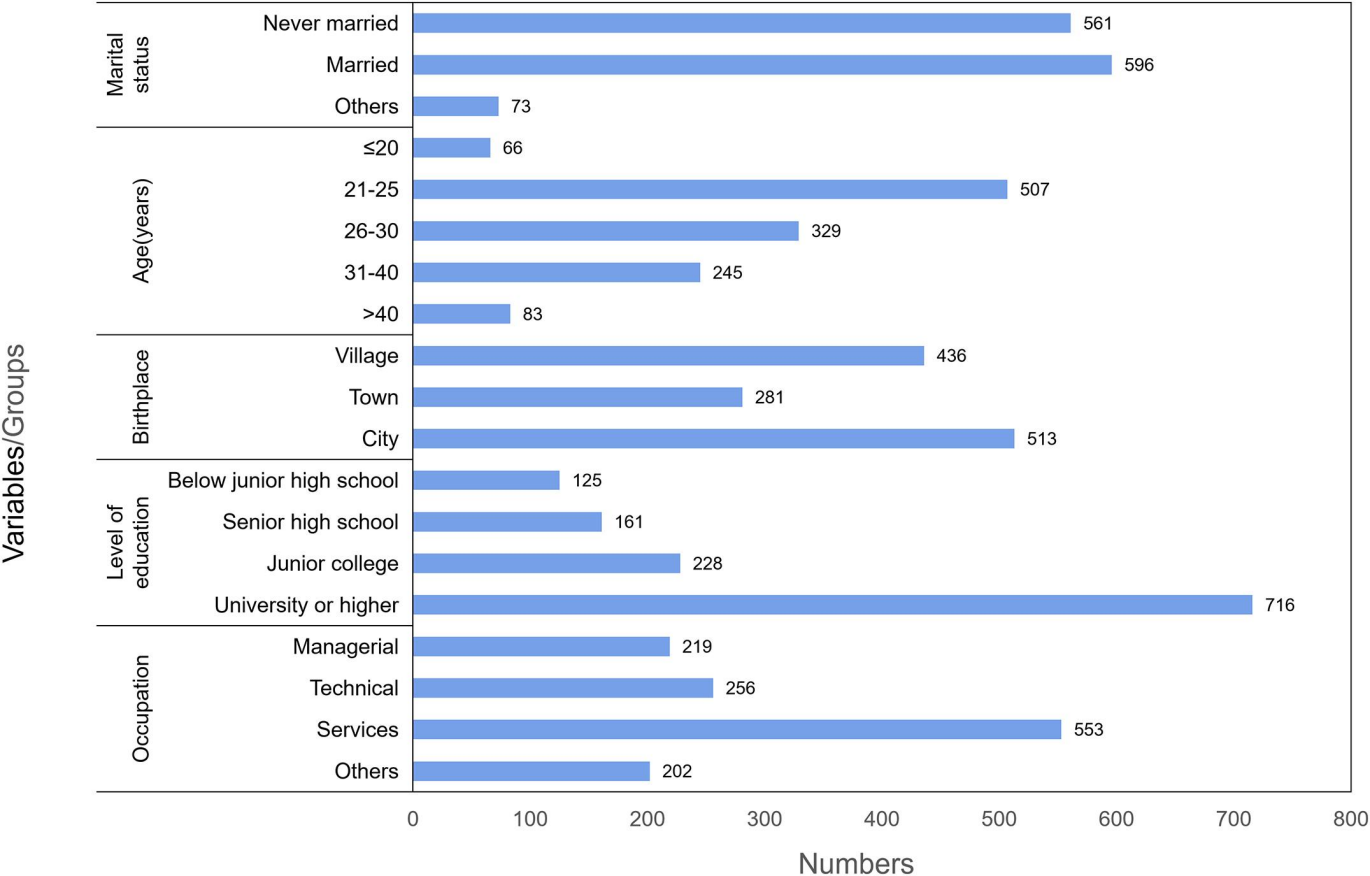
Basic subject information.

A total of 1,289 women aged 15–44 years were randomly selected, and 1,230 women completed the survey (response rate = 95.4%). Among the respondents , 596 (48.5%) were married, and 513 (41.7%) resided in urban areas. More than two-fifths (507; 41.2%) of participants were aged between 21 and 25 years. Over half of the population (716; 58.2%) had a college degree or higher. In terms of occupation, the largest proportion of respondents (553; 45%) worked in the service industry, with managerial, technical, and others occupations accounting for 17.8%, 20.8%, and 16.4%, respectively (Figure 1).

### Demographic distribution of unwanted pregnancy

**Figure 2:**
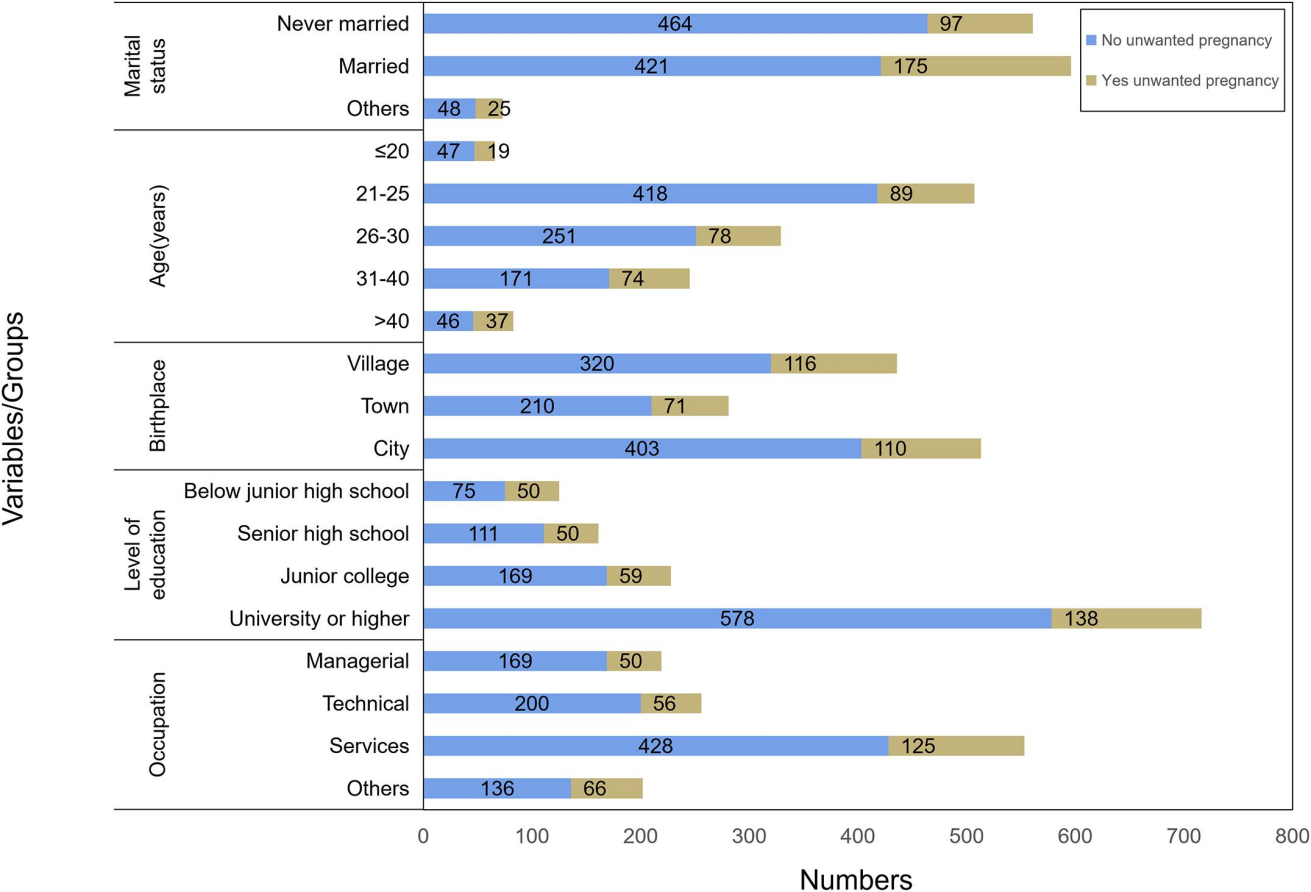
Relationship between sociodemographic variables and unwanted pregnancy.

Figure 2 summarizes the proportion of unwanted pregnancy across the included sociodemographic characteristics. Among the respondents, 297 (24.1%) reported experiencing an unwanted pregnancy. More than half of unwanted pregnancies occurred among married women (175; 58.9%), whereas the rate of unwanted pregnancy among unmarried, divorced, and widowed women gradually decreased to 32.7%, 7.1%, and 1.3%, respectively. Women aged 40–44 years had a higher proportion of unwanted pregnancy (44.6%), whereas the lowest proportion was recorded among women aged 21–25 years (17.6%). The rate of unwanted pregnancy among urban residents was 21.4%, whereas it was 26.6% occurred among women from rural areas. Approximately 45% of unwanted pregnancies occurred among women with a primary level of education, whereas women with a higher educational level reported a lower prevalence. Women with secondary education reported 31.1% of unwanted pregnancies, whereas women with a college or higher level of education reported 19.3%. Approximately four in 10 unwanted pregnancies (42%) occurred among women from the service industry.

### Reasons and outcomes of unwanted pregnancy

**Figure 3:**
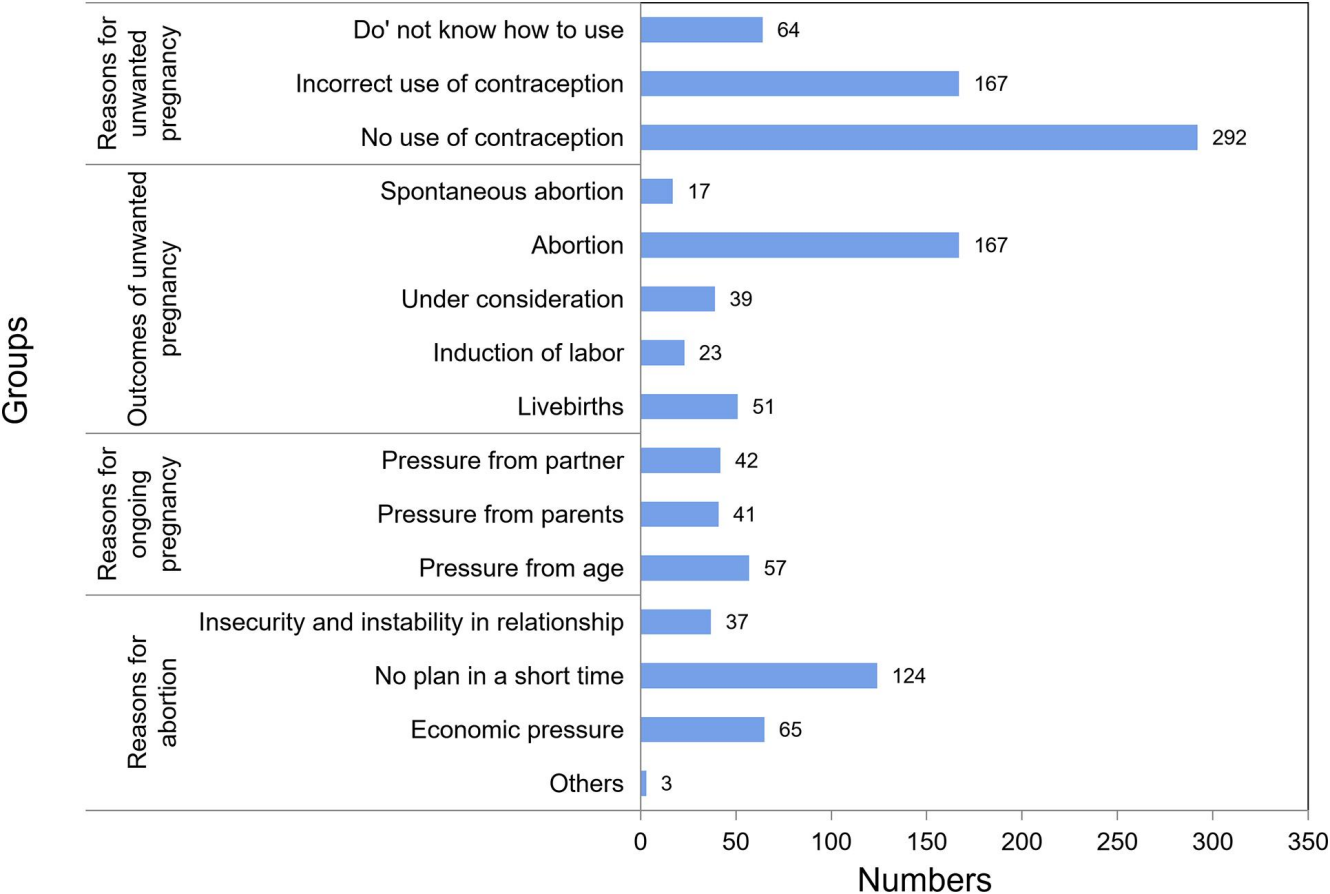
Reasons and outcomes of unwanted pregnancy.

The most common reason for unwanted pregnancy was unprotected intercourse, followed by incorrect use of contraception and lack of knowledge about contraceptive methods. Approximately half of unwanted pregnancies were terminated by means of abortion, primarily due to lack of short-term family planning or economic pressure. Less than 8% of women chose to give birth, and three out of five had given birth because of external pressure (parents or partners) (Figure 3).

### Logistic regression results of unwanted pregnancy

**Figure 4:**
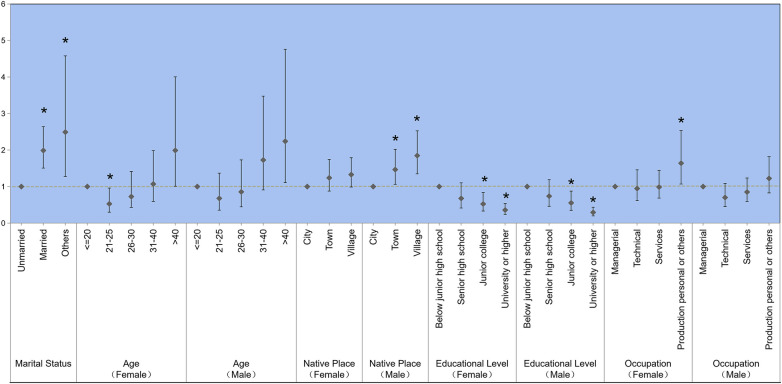
Univariate hierarchical logistic regression analysis of unwanted pregnancy (*p < 0.05).

Figure 4 presents the results of the univariate logistic regression analysis. Divorced and widowed (others) women had significantly higher odds of unwanted pregnancy (OR = 2.491; 95% CI = 1.449–4.204), followed by married women (OR = 1.998; 95% CI = 1.504–2.64), compared with the unmarried reference group. Higher odds of unwanted pregnancy occurred among women aged >40 years (OR = 1.990; 95% CI = 1.010–4.006), whereas women aged 21–25 years had fewer odds of unwanted pregnancy (OR = 0.527; 95% CI = 0.299–0.959). Compared with women educated below junior high school level, those with junior college (OR = 0.524; 95% CI = 0.329–0.834) or university or higher education (OR = 0.358; 95% CI = 0.240–0.538) had lower odds of unwanted pregnancy. Women in other occupations (agriculture and forestry positions) had higher odds of reporting unwanted pregnancy compared with those in managerial positions (OR = 1.640; 95% CI = 1.068–2.533). Women whose male partners were from towns (OR = 1.465; 95% CI = 1.060–2.021) and villages (OR = 1.848; 95% CI = 1.351–2.527) had higher odds of unwanted pregnancy compared with those whose male partners were from the city. Women whose male partners had junior college (OR = 0.553; 95% CI = 0.347–0.877) or university-level or higher education (OR = 0.293; 95% CI = 0.197–0.436) had significantly lower odds of unwanted pregnancy compared with women whose male partners had less than junior high school education (Figure 4).

Multivariate logistic regression analysis was used to control the influence of all the above-mentioned factors on the results. After adjusting for relevant factors, similar results were observed. The results indicated that married women had significantly higher odds of unwanted pregnancy (aOR = 1.665; 95% CI = 1.133–2.456) compared with unmarried women. Women aged 21–25 years had a lower risk of unwanted pregnancy compared with women aged <20 years (aOR = 0.528; 95% CI = 0.291–0.987). Women with a university-level education or higher were less likely to experience unwanted pregnancy compared with those with less than junior high education (aOR = 0.555; 95% CI = 0.352–0.881). Women with male partners from villages had higher odds of unwanted pregnancy compared with those with male partners from the city (aOR = 1.455; 95% CI = 1.042–2.027). It was also interesting to note that women whose male partners had junior college (aOR = 0.603; 95% CI = 0.370–0.984) or university-level or higher education (aOR = 0.384; 95% CI = 0.248–0.596) had lower odds of unwanted pregnancy than women whose male partners had less than junior high school education (Table 1).

**Table 1:** Multivariate hierarchical logistic regression analysis of unwanted pregnancy.

Variables	Groups	Unwanted pregnancy	*β*	S.E.	*Z* value	*p*-Value	OR (95% CI)
Marital status	Unmarried	97					1
	Married	175	**0** **.** **510**	**0** **.** **197**	**2** **.** **588**	**0** **.** **010**	**1.665 (1.133–2.456)**
	Others	25	0.388	0.320	1.215	0.224	1.474 (0.780–2.737)
Female age (years)	≤20	19					1
	21–25	89	**−0** **.** **639**	**0** **.** **310**	**−2** **.** **060**	**0** **.** **039**	**0.528 (0.291–0.987)**
	26–30	78	−0.565	0.345	−1.636	0.102	0.568 (0.291–1.133)
	31–40	74	−0.364	0.355	−1.027	0.305	0.695 (0.350–1.410)
	>40	37	0.132	0.394	0.335	0.738	1.141 (0.530–2.493)
Female birthplace	City	110					1
	Town	71	0.216	0.183	1.181	0.238	1.241 (0.865–1.772)
	Village	116	0.244	0.161	1.517	0.129	1.276 (0.931–1.748)
Female level of education	Below junior high school	50					1
	Senior high school	50	−0.292	0.258	−1.133	0.257	0.747 (0.450–1.238)
	Junior college	59	−0.367	0.255	−1.442	1.149	0.693 (0.421–1.143)
	University or higher	138	**−0** **.** **589**	**0** **.** **233**	**−2** **.** **522**	**0** **.** **012**	**0.555 (0.352–0.881)**
Female occupation	Managerial	50					1
	Technical	56	0.186	0.229	0.811	0.417	1.204 (0.770–1.615)
	Services	125	0.074	0.204	0.365	0.715	1.077 (0.726–1.615)
	Production personal or others	66	0.408	0.232	1.758	0.079	1.503 (0.956–2.375)
Male age (years)	≤20	13					1
	21–25	70	−0.258	0.360	−0.716	0.474	0.773 (0.390–1.615)
	26–30	70	−0.102	0.365	−0.281	0.779	0.903 (0.451–1.901)
	31–40	99	0.554	0.357	1.550	0.121	1.740 (0.884–3.620)
	>40	45	0.593	0.385	1.540	0.124	1.809 (0.866–3.950)
Male birthplace	City	111					1
	Town	85	0.330	0.175	1.891	0.059	1.391 (0.987–1.958)
	Village	101	**0** **.** **375**	**0** **.** **170**	**2** **.** **210**	**0** **.** **027**	**1.455 (1.042–2.027)**
Male level of education	Below junior high school	55					1
	Senior high school	55	−0.280	0.252	−1.110	0.267	0.756 (0.461–1.238)
	Junior college	58	**−0** **.** **505**	**0** **.** **249**	**−2** **.** **026**	**0** **.** **043**	**0.603 (0.370–0.984)**
	University or higher	129	**−0** **.** **957**	**0** **.** **223**	**−4** **.** **287**	**<0** **.** **001**	**0.384 (0.248–0.596)**
Male occupation	Managerial	57					1
	Technical	45	−0.059	0.237	−0.248	0.804	0.943 (0.592–1.499)
	Services	111	−0.130	0.201	−0.648	0.517	0.878 (0.593–1.307)
	Production personal or others	84	0.084	0.212	0.398	0.691	1.088 (0.719–1.652)

The bolded values indicate statistically significant differences.

### Contraceptive needs

**Figure 5:**
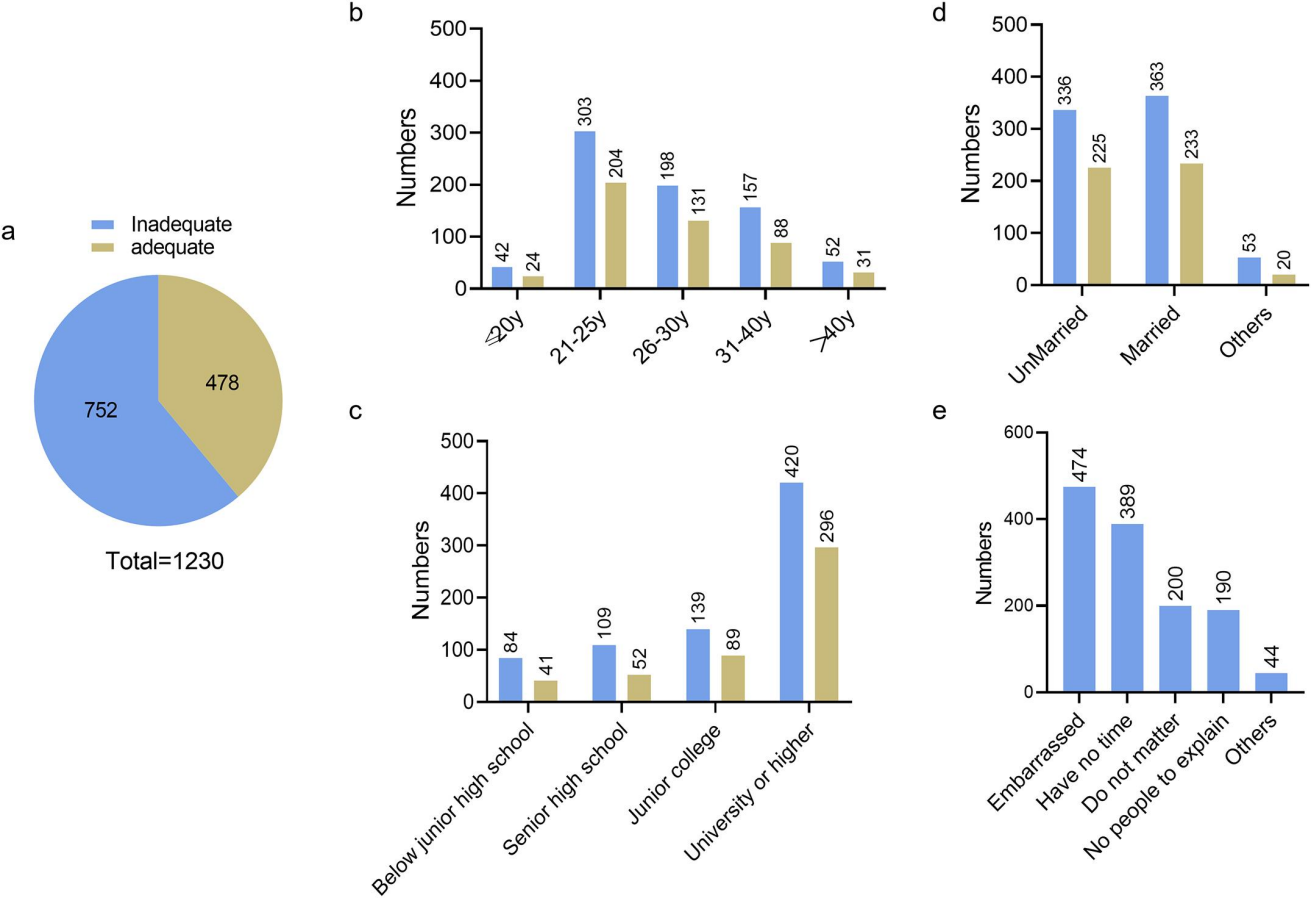
Number of women receiving adequate/inadequate contraceptive education: **(a)** total; and **(b–e)** proportions according to age, education level, and marital status, respectively.

Figure 5 illustrates the contraceptive needs of the respondents. Among the 1,230 women surveyed, nearly 61% did not receive adequate contraceptive education. When grouped by age, the 21–25-year age group accounted for the largest proportion. In terms of educational level, the proportions decreased as the level of education increased. With regard to marital status, unmarried women had the highest proportion of unmet contraceptive education needs. The three main reasons for the lack of contraceptive education were embarrassment (53.1%), lack of time (43.7%), and indifference (“don't care”) (22.3%) (Figure 5).

**Figure 6:**
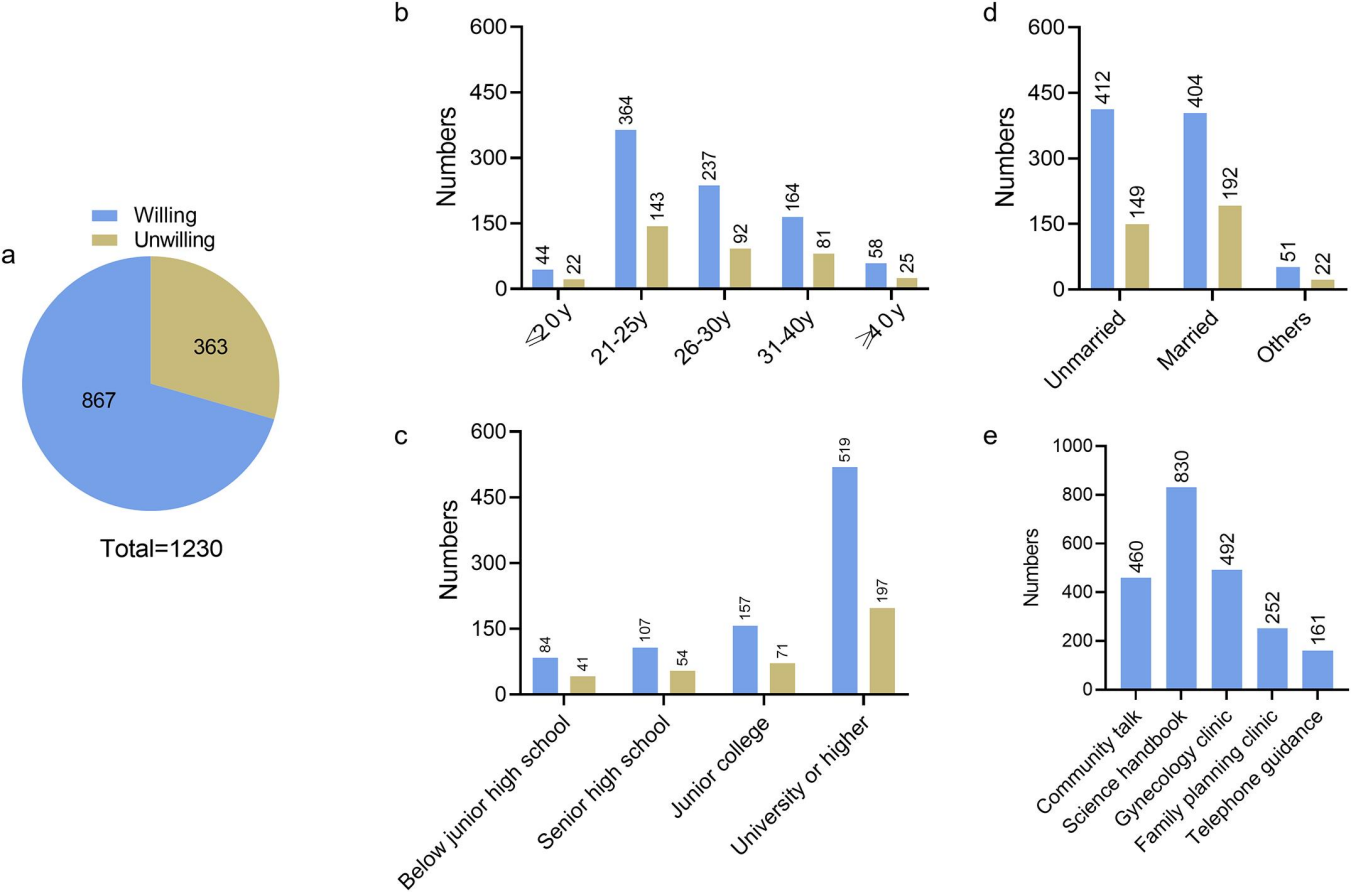
Number of women willing/not willing to receive contraceptive instructions: **(a)** total; and **(b–e)** proportions according to age, education level, and marital status, respectively.

The Internet was the most common source of contraceptive education (65.6%; 392/598), followed by hospitals (60.4%; 391/598) and schools (57.2%; 342/598). However, 70.4% (867/1,230) of respondents were very willing to accept professional contraceptive guidance. Women aged 21–30 years, those with a college education or higher, and unmarried women were more likely to receive contraceptive guidance. The most acceptable way to access contraceptive guidance was through the use of science manuals (67.48%; 830/1,230), followed by gynecological specialist clinics (40%; 492/1,230) and community lectures (37.24%; 458/1,230) (Figure 6).

## Discussion

Unwanted pregnancy remains a significant public health concern worldwide (3). Although evidence suggests that rates of unwanted pregnancy have declined worldwide due to increasing access to and use of contraception, approximately half of all pregnancies are still unintended (7). Chengdu is a newly developing first-tier city in China with a large number of young residents. Currently, the focus in this city is to improve post-abortion care services in hospitals. However, this approach overlooks individuals who are sexually active but have not yet experienced an unwanted pregnancy. Moreover, women who seek induced abortion at public hospitals are the primary target group for post-abortion care, but many others undergo self-induced drug abortion or spontaneous abortion in private clinics. Consequently, some abortions occurring outside the organized health sector are not captured. Without statistical data on these groups, it is difficult to fully understand the status of unwanted pregnancy or the contraceptive needs of women of childbearing age in the city. In this study, we took the community as the unit and surveyed all women of childbearing age in the five main districts. We used a self-administered questionnaire to understand the current situation of unwanted pregnancy, implement interventions, prevent unwanted pregnancy at an early stage, and ultimately reduce its incidence in Chengdu.

Our study indicates that being married was associated with an increased risk of unwanted pregnancy. This result corroborates the findings of previous studies, such as the study by Nyarko et al. (8), which found that married women are more likely to experience unwanted pregnancy than those who never married or those who were previously married. Possible explanations include non-use of contraceptives or contraceptive failure, as married women may believe that contraceptive use has serious side effects and some may view it is a sin. Another important explanatory factor may be gender inequality within couples (9). Condom use is often determined by men (10). Moreover, an increased risk of unwanted pregnancy may occur when male partners hold economic power and refuse to use condoms (11). In our view, unwanted pregnancy can occur easily due to improper or non-use of contraception: Consistent and effective use of contraception during every act of intercourse is required for married couples to avoid unwanted pregnancy, but it appears to be challenging for many couples to achieve. Therefore, it is necessary to further strengthen contraception awareness among couples, with family planning playing a more significant role.

According to the findings of this study, age plays a significant role in the risk of unwanted pregnancy. Women in other age categories were more likely to experience unwanted pregnancy compared with those aged 21–25 years. This finding contradicts previous studies that found women aged 20–34 years to have the highest proportion of unwanted pregnancy (12); other studies have also found that girls aged 15–19 years are more likely to experience unwanted pregnancy compared with women in other age categories (13). Previous research has attributed the higher risk of younger women to higher fertility levels, more frequent sexual activity, reluctance to seek family planning advice from relatives or family care organizations, and higher rates of contraceptive failure, all of which contribute to unwanted pregnancy. From our perspective, however, education acted as a powerful individual predictor of behavior. Most women in the 21–25-year age group were still in school and were more willing and able to obtain contraceptive knowledge, leading to a lower rate of unwanted pregnancy. Furthermore, most women aged 21–25 years were unmarried, with partners who were boyfriends who did not live with them, which also aligns with the findings mentioned earlier that married women have a higher rate of unwanted pregnancy.

Moreover, the risk of unwanted pregnancy was found to be predicted by the woman's level of education. Data in the present study showed that, compared with the women with no formal education, women with a college degree or higher—or those whose male partner had a college degree or higher—were less likely to experience unwanted pregnancy. Our findings are consistent with those of other studies reporting that women whose husbands had with no education or only primary education were significantly more likely to experience unwanted pregnancy (14, 15). Education has the potential to raise women's awareness of the implications of unwanted pregnancy and the contraceptive methods available, which educated women are more likely to utilize (16). Font-Ribera et al. (17) also noted that women with some formal education are more empowered to take charge of their sexual and reproductive health matters compared with women without formal education. Furthermore, education has also been shown to empower women through career opportunities that often lead to higher socioeconomic status, which in turn may increase their autonomy and ability to negotiate safer sex practices, including condom use (18). Moreover, the birthplace of the male partner is significantly associated with unwanted pregnancy among Chengdu women. The data indicate that women whose male partners were from villages were 1.5 times more likely to experience unwanted pregnancy than those whose partners were from the city. Male partners from villages may have low socioeconomic status and may perceive a pregnancy as unintended if they feel that they cannot support the unborn child financially.

The desire to avoid pregnancy does not always translate into contraceptive use (19). High rates of unwanted pregnancy may also be attributable to limited reproductive health education and knowledge. Gaps in contraceptive knowledge directly affect the choice of contraceptive method and the effectiveness of contraception (20). In our study, more than half of the women had never received contraceptive guidance or related education. Furthermore, as the level of education decreased, the unmet need for contraception increased. Approximately 70% of the respondents obtained contraceptive education from the Internet rather than from family planning consultation clinics or gynecology-related clinics. It should be noted out that more than two-thirds of the respondents said “yes” when asked whether they would be willing to receive contraceptive education, with the largest groups being unmarried women, those aged 21–30 years, and those with a college education or higher. Meanwhile, popular science handbooks, gynecological clinics, and community-based initiatives were deemed the most acceptable methods of accessing contraceptive information.

## Conclusion

The current situation regarding unwanted pregnancy among women of childbearing age in Chengdu is a matter of concern. Several factors—including the age, marital status, and educational level of the women themselves, as well as the educational level and place of birth of their sexual partners—contribute to this issue. There is an urgent need to address the demand for contraceptive guidance among women. The first step should be to promote comprehensive contraceptive knowledge throughout the city. Furthermore, it is important to emphasize that contraception is not the sole responsibility of women, and men must also play an active and informed role in contraceptive decision-making. This study has several limitations. First, Chengdu has many districts, but we focused only on five major districts, which limits the generalizability of our findings. Therefore, a larger sample size is required. Second, we should use separate questionnaires for men in order to better reflect the role of men in unwanted pregnancy. Third, some questions in the questionnaire need to be more clearly worded to obtain more accurate data.

## Data Availability

The original contributions presented in the study are included in the article/Supplementary Material, further inquiries can be directed to the corresponding authors.
